# Modulation of Autophagy in Adrenal Tumors

**DOI:** 10.3389/fendo.2022.937367

**Published:** 2022-07-28

**Authors:** Diana Sousa, Sofia S. Pereira, Duarte Pignatelli

**Affiliations:** ^1^i3S - Instituto de Investigação e Inovação em Saúde, Universidade do Porto, Porto, Portugal; ^2^Cancer Signaling & Metabolism Group, IPATIMUP- Institute of Molecular Pathology and Immunology of the University of Porto, Porto, Portugal; ^3^Unidade Multidisciplinar de Investigação Biomédica (UMIB), Instituto de Ciências Biomédicas Abel Salazar (ICBAS), Universidade do Porto, Porto, Portugal; ^4^ITR - Laboratory for Integrative and Translational Research in Population Health, Porto, Portugal; ^5^Department of Endocrinology, Centro Hospitalar e Universitário de S. João, Porto, Portugal; ^6^Department of Biomedicine, Faculty of Medicine of the University of Porto, Porto, Portugal

**Keywords:** autophagy, adrenal gland, adrenal tumors, adrenocortical carcinoma (ACC), pheochromocytoma (Pheo)

## Abstract

Adrenal masses are one of the most common tumors in humans. The majority are benign and non-functioning and therefore do not require immediate treatment. In contrast, the rare adrenal malignant tumors are often highly aggressive and with poor prognosis. Besides usually being detected in advanced stages, often already with metastases, one of the reasons of the unfavorable outcome of the patients with adrenal cancer is the absence of effective treatments. Autophagy is one of the intracellular pathways targeted by several classes of chemotherapeutics. Mitotane, the most commonly used drug for the treatment of adrenocortical carcinoma, was recently shown to also modulate autophagy. Autophagy is a continuous programmed cellular process which culminates with the degradation of cellular organelles and proteins. However, being a dynamic mechanism, understanding the autophagic flux can be highly complex. The role of autophagy in cancer has been described paradoxically: initially described as a tumor pro-survival mechanism, different studies have been showing that it may result in other outcomes, namely in tumor cell death. In adrenal tumors, this dual role of autophagy has also been addressed in recent years. Studies reported both induction and inhibition of autophagy as a treatment strategy of adrenal malignancies. Importantly, most of these studies were performed using cell lines. Consequently clinical studies are still required. In this review, we describe what is known about the role of autophagy modulation in treatment of adrenal tumors. We will also highlight the aspects that need further evaluation to understand the paradoxical role of autophagy in adrenal tumors.

## 1. Adrenal Tumors

Adrenal tumors are common endocrine alterations in the normal adrenal gland histology. Radiological studies have demonstrated that the prevalence of adrenal tumors in the general population is 2-4%, but these numbers go up to > 10% in subjects aged >70 (1). The majority of adrenal tumors are benign non-functioning masses. However, two different malignancies can originate primarily from the adrenal gland: adrenocortical carcinomas (ACC) arising from the adrenal cortex and pheochromocytomas (PCC) arising from chromaffin cells of the adrenal medulla. Similar tumors may originate from extra-adrenal paraganglia and are called Paragangliomas. Pheochromocytomas and Paragangliomas together are sometimes designated as PPGLs (2).

In the present review, we will focus on the tumors originated primarily in adrenal gland, and therefore we will limit our analysis to reports which studied ACC or PCC only. Importantly, the terms “malignant” PCC and “benign” PCC were retracted in the 2017 WHO classification of endocrine tumors, because there is no histological system that is currently recommended for the biological aggressiveness assessment of this group of tumors. Thus, all PCCs may have metastatic potential (3).

Both ACC and PCC are rare and ACC usually have an aggressive behavior and a poor prognosis due to the difficulties to establish an early diagnosis. Indeed, ACC diagnosis usually occurs in an advanced clinical stage (ENS@T stage IV), which is characterized by a 5-year overall survival less than 15% (4). The prognosis of metastasized PCC is also generally poor, related with local recurrence or metastasis proliferation, presenting a heterogeneous 5-year survival rate varying between 40% and 70% (5, 6). The above mentioned data stresses the importance of the improvement in diagnostic tools of adrenal tumors.

Regarding ACC treatment, surgery with complete resection is the only treatment with curative potential. In most patients with locally advanced and metastatic ACC (stages III and IV), first-line therapy with mitotane alone or mitotane plus etoposide, doxorubicin and cisplatin (EDP-M) are the recommended options. Loco-regional therapies, namely radiotherapy, may be adopted in addition to systemic therapies in selected patient populations (2).

Currently, mitotane is the only approved drug for the treatment of ACC patients in monotherapy. However different studies have been showing its moderate long-term efficacy, also reporting severe adverse effects and toxicity in some cases, as well as drug interactions (7).

In PCC patients, surgery should also always be considered and other treatment choices include loco-regional therapies, radiopharmaceutical agents, systemic chemotherapy and molecular-targeted therapies. Molecular targeted therapies that involved everolimus, imatinib or sunitinib have been used, with dissimilar results (8).

Research about the impact of autophagy in adrenal tumors is recent and usually focuses on the role of autophagy in the context of the mechanism of action of pharmacological treatments. In the following section, we will briefly overview the autophagic process highlighting the key players, and then explore the modulation of autophagy in the context of malignant adrenal tumors.

## 2. Autophagic Process: Pro-Death or Pro-Survival Mechanism? A Matter of Context

In 1963, Christian de Duve proposed the concept of autophagy for the first time, winning the Nobel Prize in Physiology, by virtue of this achievement, eleven years later (9). Since then, several studies aiming to investigate this process were performed and currently it can be claimed that almost all the basic functions of the cell are somehow associated with autophagy. The main result of the activation of this process is the lysosome-mediated degradation of cellular components (10).

Autophagy is a highly dynamic, multi-step process. This mechanism initiates in the cytoplasm by the formation and expansion of a double-membrane structure, the phagophore, which elongates into an autophagosome. Subsequently, the scission of the intraphagophore membrane and the fusion of the outer autophagosomal membrane with lysosomes takes place. This fusion of the autophagosome with a lysosome originates the autophagolysosome, where the cargo is degraded by lysosomal hydrolases under acidic pH (11).

**Figure 1:**
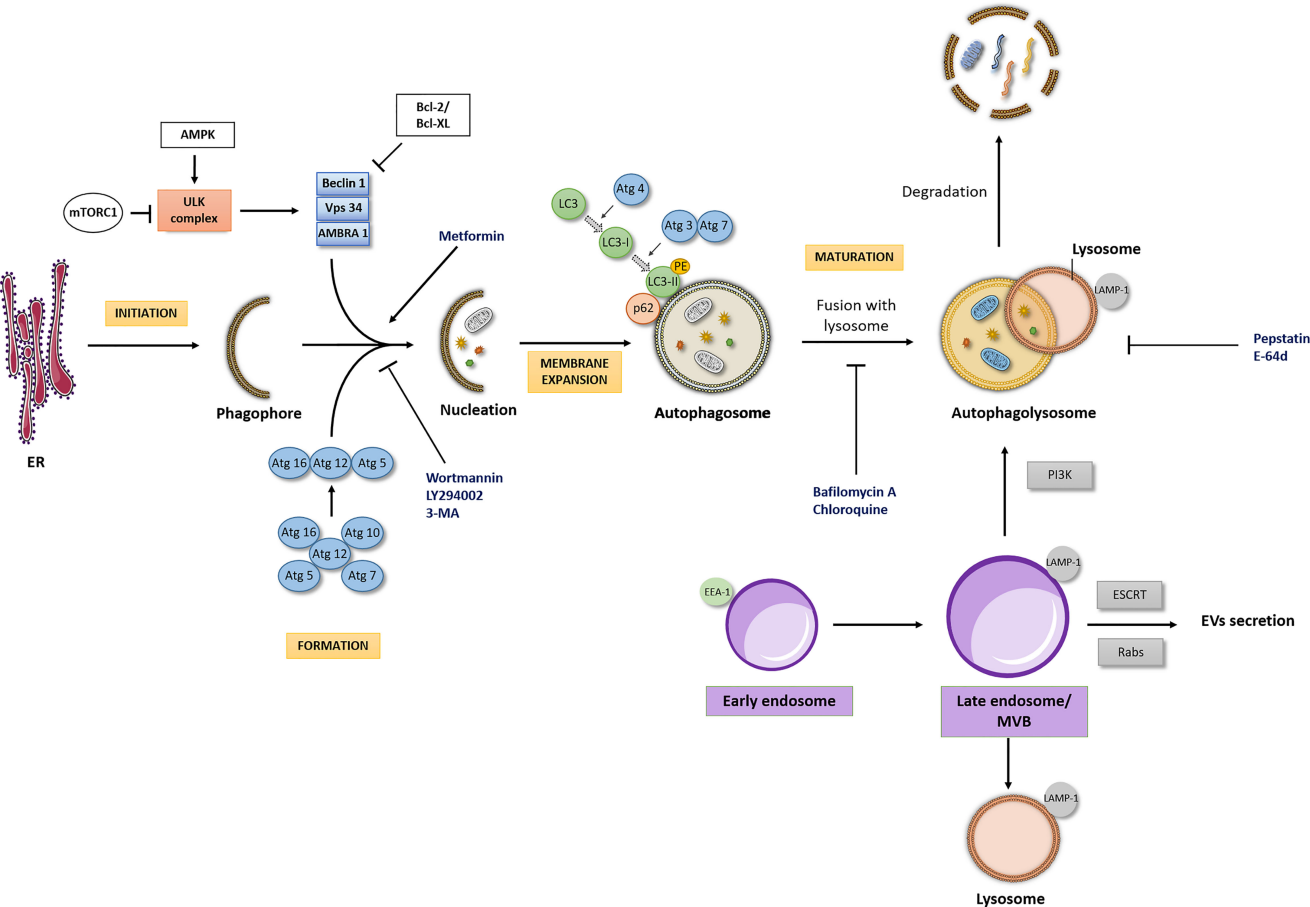
Autophagic flux and interdependency of the intracellular pathways. Autophagic Flux: The four major stages of the autophagic pathway are shown in yellow boxes (initiation, formation, membrane expansion and maturation). Autophagy is a highly regulated process, focused on the lysosomal degradation of cellular components and/or recycling of cytosolic compounds. Initiation of formation of the phagophore begins when the ULK kinase complex is activated. A dedicated cohort of ATG proteins assemble into functional complexes, which are activated and recruited to membranes to initiate autophagy. The lipidation of ATG8/microtubule- associated protein 1 light chain 3 (LC3), promoting the conversion of LC3-I into LC3-II in membrane expansion stage and the degradation of p62/SQSTM1 (Sequestosome 1) in maturation stage are pivotal steps of autophagic process. Importantly, autophagic flux may be modulated by different drugs (here represented at blue font). PE, phosphatidylethanolamine; ER, Endoplasmic reticulum. Interdependency of the intracellular pathways: The endocytic pathway seems to be the scaffold for other intracellular pathways, such as the recycling, lysosomal and the autophagic pathway, with the endosome (here represented in purple boxes) being the main responsible for the interplay between these pathways. The interdependency of those intracellular pathways influences a series of cellular processes and consequently its deregulation may cause different pathologies, including cancer. LAMP-1, Lysosome-associated membrane glycoprotein- marker of late endosome/lysosome; EEA1, Early Endosome Antigen 1- typical marker of early endosome.

The autophagic process can be modulated at several steps, both positively and negatively (**Figure 1**). The initiation of formation of the phagophore begins by the activation of the Unc-51-Like Kinase (ULK) complex. Briefly, after a strong stimulus such as nutrient starvation or under other stress conditions, the mammalian target of rapamycin (mTOR) is inhibited, which initiates the formation of the ULK complex. Importantly, the molecular regulation of autophagy machinery also occurs by mTOR-independent pathways (12). The activation of the ULK complex corresponds to its translocation at a discrete location on the endoplasmic reticulum that has been marked by ATG9 (13). Then, the ULK complex activates other players, including Vacuolar Protein Sorting (VPS34), Activating Molecule in Beclin-1- Regulated Autophagy (AMBRA1) and Beclin-1 (14). Following this, the elongation and expansion of the phagophore membrane is an important stage in autophagosome formation. This process is modulated by two inter-related systems: one which conjugates ATG12 to ATG5 in association with ATG16L1 and other system which drives the lipidation of the ATG8 family members (15). In ATG12-ATG5-ATG16 system, ATG12 is initially triggered by the ATG7 in an ATP-dependent way. The second ubiquitin-like system induces the conjugation of phosphatidylethanolamine to ATG8/microtubule- associated protein 1 light chain 3 (LC3) regulated by ATG4, ATG7 and ATG3. This lipidation which converts LC3-I into LC3-II is a crucial step of autophagy. Indeed, ATG8 and ATG8-family proteins are the most widely monitored autophagy-related proteins (16). The maturation of autophagosome is regulated by Lysosome-associated membrane glycoprotein (LAMP) 1/2.

Following autophagosome formation, as referred above, occurs its fusion with lysosome originating the autophagolysosome, where the cargo is degraded and/or recycled. The formation of autophagolysosome is regulated by cytoskeleton components and related motor proteins, tethering factors, phospholipids, and specific SNARE complexes (13). Importantly, a pivotal player which binds directly LC3 is the p62/SQSTM1 (Sequestosome 1). P62/SQSTM1 is an autophagy receptor that links ubiquitinated proteins to LC3 promoting their degradation. Interestingly, p62/SQSTM1 is itself degraded in the final steps of autophagic process (17).

In the reports aiming to unravel the role of autophagy in different contexts, the increased levels of LC3-II are usually presented as a marker of autophagy induction. Nevertheless, it is important to refer that an increase in LC3-II expression could reflect either induction or inhibition of autophagy since a reduction in autophagosome turnover will also result in LC3-II increase (18–20). Therefore, as highlighted in the recent edition of the “Guidelines for the use and interpretation of assays for monitoring autophagy (4^th^ edition)”, the evaluation of LC3-II expression must be complemented by assays to estimate overall autophagic flux, such as co-treatment with known autophagic modulators, in order to obtain a correct interpretation of the results (16). The concomitant evaluation of p62/SQSTM1 levels is also performed since this protein accumulates in cells when autophagy is inhibited.

Autophagy modulation has been explored in both physiological and pathological conditions (21). In cancer, the effect of autophagy has been referred as a “double-edged sword’ due to its role in both tumor progression and tumor suppression (22). For example, suberoylanilide hydroxamic acid was shown to induce autophagic cell death and significantly reduced the growth of breast cancer cells resistant to tamoxifen (23). By contrast, autophagy induced by different chemotherapeutics, such as paclitaxel, tamoxifen, epirubicin and trastuzumab was associated with a drug-resistant phenotype (24–27).

The cross-talk between apoptosis and autophagy described in different tumor models is another important feature that should be taken in consideration. Apoptosis is the canonical programmed cell death pathway (28). However, as described above, the autophagic cell death have also been reported in different cancers. The interplay between apoptosis and autophagy occurs through the regulation by proteins which are able to modulate both processes, such as: i) Beclin-1 which interacts with the anti-apoptotic B-cell lymphoma 2 (BCL-2) proteins, ii) ATG5 which interacts with Fas-associated protein with death domain (FADD) or iii) p53 which interacts with mTOR, among other players. This cross-talk was elegantly reviewed by Minfei Su et al. (29).

## 3. Expression of Autophagic Key Players in Adrenal Tumor Tissues

The number of studies that evaluated the levels of important modulators of autophagy in tumoral adrenal samples is low. A recent study analyzed the expression of autophagy players in adrenal tumors from patients, by immunohistochemistry, and integrated these results with the patients’ clinicopathologic parameters (30). Tissue microarray was performed in 115 cases of adrenocortical adenoma (ACA), 17 ACC cases and 189 PCC. This analysis revealed differences in the expression of autophagy-related proteins according to adrenal gland tumor type. In PCC, the proportion of LC3A, LC3B and Beclin-1 positivity was found to be higher when compared with adrenocortical tumors (ACT), whereas p62 positivity was found to be lower in PCC. Notably, p62 positivity was shown to be predictive of shorter overall survival in patients with PCC, by multivariate analysis. Among the two types of ACT, the proportion of positive LC3B in isolated single positive cells was higher in ACC than in ACA. Interestingly, a study from 2016, evaluated the expression of Beclin-1 in ACC tumors and in normal adrenocortical tissue. Immunohistochemistry of 35 ACC tissues and 15 normal tissues showed that the positive expression of Beclin-1 in ACC tissues was significantly lower when compared with normal tissues. These results were further validated (in 15 ACC samples and 15 normal samples) by western blot (31). Furthermore, negative expression of Beclin-1 was significantly correlated with advanced stage, regional lymph, node metastasis, increasing T stage, and poor differentiation. Those two studies apparently show contradictory results. In the first report, the malignant phenotype of adrenal tumors seems to be associated with an induction of autophagy whilst in the second report this phenotype was associated with reduced expression of a key positive regulator of autophagic process. However, a meticulous evaluation of these findings is required. As highlighted in the recent edition of the “Guidelines for the use and interpretation of assays for monitoring autophagy (4^th^ edition)”, it could be difficult to clearly monitor autophagy in tissues of formalin-fixed and paraffin-embedded biopsy samples, because i) tissues fixed in formalin have low or no LC3 detectable by routine immunostaining and ii) phospholipids melt together with paraffin during the sample preparation (16). Moreover, the evaluation of autophagy only in paraffin blocks is not considered a robust approach since a detailed investigation of the autophagic flux is also required (as described above in section 2). Immuno-electron microscopy could be a valuable tool in this evaluation, nevertheless, as described for immunohistochemistry technique, a proper fixation is also essential. In addition, complementary *in vitro* experiments such as immunofluorescence for key autophagy regulators and the use of autophagy modulators commercially available may provide better insights into the role of autophagy in adrenal gland tumors, namely in tumorigenesis, tumor progression and tumor metastasis.

## 4. Unravelling the Dual Role of Autophagy in Adrenal Tumors Using *In Vitro* Models

A suitable *in vitro* model for the study of adrenal tumors should be chosen taking into consideration the aim of the analysis. For adrenocortical studies, the most used cell lines are H295-derived cell strains and SW13. However, some diverging results have been obtained using those cell lines. It has been shown that culture conditions (such as culture serum, lipoproteins and BSA concentration or culture passages) are of outmost importance for reproducibility. In addition, it is important to refer that features of each cell line, H295 and SW13, are different. Due to the complex role of autophagy, considering these features is essential to obtain solid conclusions. SW13 cells were isolated and amplified from a 55-year-old female with a small cell type carcinoma excised from the adrenal cortex (32). Due to their atypical histology and absence of steroidogenic potential, it is unclear whether SW13 cell lines are primary adrenocortical carcinoma or resulting from adrenal cortex metastases (33). By contrast, H295 cell line was established from a female patient with a steroid secreting ACC whose tumor was extracted, defragmented, and maintained in culture media for one year (34). Due to poorly adherent phenotype of NCI-H295 cells, three H295R sub-strains were generated, presenting a tightly adherent phenotype and a reduction in doubling time. Importantly, these strains are capable of producing each of the zone-specific steroids.

In addition, Y1 cell line derived from the cortex of the adrenal gland from *Mus Musculus* is also used in a small scale in autophagy studies.

Regarding PCC cell line models, PC12 cells are widely used, however the results should be carefully analyzed since he PC12 cell line was derived from a transplantable rat PCC and some authors argue that these cells do not reflect exactly the pathogenesis of malignant cells.

### 4.1 Pro-Survival Role of Autophagy in Adrenal Tumors and Its Impact in Adjuvant Therapy

In 2014, Sbiera et al. claimed that the adrenal specific toxicity of mitotane was related with the promotion of endoplasmic reticulum (ER) stress (35). In this study, the mitotane treatment triggered apoptosis and decreased the synthesis of steroid hormone in an ACC cell line. The authors hypothesized that the intracellular overload of sterols induced by mitotane treatment triggered the canonical ER-stress pathway which eventually would lead to increased apoptosis and decreased steroidogenesis. Importantly, unsupervised pathway analyses confirmed that one of the most significantly altered pathways by mitotane was the ER-stress response pathway. In addition, salubrinal (an ER-stress inhibitor) partly reversed mitotane-induced expression of ER-stress marker C/EB Homologous Protein (CHOP) whilst thapsigargin (an ER-stress inducer) increased the mitotane effect in NCI-H295 cells. Some years later, Tompkins K. et al. described that mitotane treatment also modulated autophagy withdrawing the toxic effects of this drug (36). Therefore, the authors suggested that inhibition of autophagy may be used as potential adjuvant therapy of mitotane treatment in patients with ACC. In this study, the authors used a cell line derived from a liver metastasis from a patient with an ACC associated to a Lynch syndrome (CU-ACC2 cell line). ER stress was induced following 6 hours of mitotane treatment while autophagy was induced only after 24 hours of drug treatment. This finding suggests that ER stress occurs prior to autophagy induction and thus, inhibition of autophagy before treatment with mitotane would improve the efficacy of the drug. Indeed, combination therapy of mitotane with chloroquine (an autophagy inhibitor) promoted higher levels of cleaved PARP (suggesting higher apoptosis induction) when compared with mitotane treatment alone.

Other recent work, also assessed the interplay between ER stress and autophagy in ACC, using a different *in vitro* model. Treatment with tauroursodeoxycholic acid (TUDCA) promoted the proliferation, migration and invasion of SW-13 cells. In addition, the authors reported that TUDCA treatment of SW13 cells also i) alleviated ER stress (through modulation of pERK/ATF4-CHOP pathway), ii) induced autophagy (evaluated by increased levels of LC3-II) and iii) inhibited the apoptosis (evaluated by altered levels of BCL-2 and Bax). Importantly, proliferation, migration and invasion studies were performed at 24 hours following TUDCA treatment while analysis of ER stress, autophagy and apoptosis was carried out upon 48 hours of treatment. These different time-points may explain the dissimilar results obtained when compared with the previous report (37). The association between ER stress and autophagy was also reported in another adrenal tumor model, PCC (38). Treatment with luteolin (an active flavonoid compound from *Lonicera japonica*) was shown to induce apoptosis through activation of endoplasmic reticulum stress sensors in PC12 cells. Moreover, the levels of LC3A were shown decreased after luteolin treatment, and so, the authors hypothesize that this compound also induced autophagy. We suggest that further experiments, namely regarding autophagic flux evaluation are required.

The inhibition of autophagy as therapeutic option was also evaluated for cisplatin treatment of ACC and the conclusions were similar to the previous studies (31). Treatment of SW13 cells with cisplatin, induced autophagy (evaluated by MDC assay, LC3-II/I ratio, and levels of Beclin-1 and p62) and induced apoptosis (evaluated by Annexin V assay). Interestingly, the concomitant treatment of cisplatin with chloroquine (autophagy inhibitor) increased the apoptosis rate of SW13 cells when compared with cisplatin monotherapy and decreased the levels of autophagic activity, as expected. Moreover, these results were confirmed *in vivo*. Concomitant treatment inhibited the growth of xenografted tumors and promoted the survival of tumor-bearing mice more efficiently than treatment with cisplatin alone.

The protective (tumor pro-survival) role of autophagy observed in the treatment of ACC with mitotane or cisplatin, was also demonstrated in the treatment of PCC using sunitinib. Sunitinib is an oral multitarget receptor tyrosine kinase inhibitor which was shown to be an active agent for the treatment of PCC. Since sunitinib targets vascular endothelial growth factor receptors (VEGFRs), it was unclear if its effect was associated only with antiangiogenic mechanism or by also direct targeting of tumor cells. Saito et al. demonstrated that treatment of PCC cells with sunitinb promoted apoptosis by inhibiting VEGFR2/Akt/mTOR/S6K1 pathways, through modulation of BCL-2 and BAD (39). Importantly, since PC-12 cells do not reflect exactly the pathogenesis of malignant cells, those results were also confirmed in SK-N-SH, a neuroblastoma cell line. Subsequently, the same group of research studied the role of autophagy in PC-12 cells treated with sunitinib (40). Indeed, the impact of mTOR pathway in autophagic flux has been described in different tumor models (41). As described in section 2, the inhibition of mTORC1 promotes the formation of the ULK complex which in turns activates the formation of autophagosome by the positive regulation of Beclin-1, Vps34 and different components of ATG family. Treatment of PC-12 cells with sunitinib significantly increased LC3-II and decreased p62 levels, indicating the induction of autophagy flux. Moreover, downregulation of ATG13 (a key protein of autophagic process) promoted an increase in apoptosis and a reduction in proliferation of PC12 cells following treatment with sunitinib. Taken together, these results suggest that inhibition of autophagy may be a promising therapeutic option for improving the anti-tumor effect of sunitinib in PCC.

### 4.2 Pro-Death Role of Autophagy in Adrenal Tumors

The aforementioned works suggested that different drugs used for ACC and PCC treatment may induce autophagy and this mechanism is responsible for some decrease in sensitivity to chemotherapy. However, as explained earlier, similar to other tumor models, in adrenal tumors autophagy may play a dual-role. The induction of autophagy as a mechanism of cell death, may be a protective mechanism in ACC. A study using rosiglitazone (RGZ) which belongs to thiazolidinediones, a class of specific peroxisome proliferator-activated receptor- γ [PPAR-γ] ligands, showed that RGZ inhibited the growth of H295R cells, activating the AMPK pathway, resulting in cellular vacuolization and enhanced autophagy. The autophagic process appeared to be independent of PPAR-γ activation and could be related to an increase in oxidative stress mediated by reactive oxygen species production with the disruption of the mitochondrial membrane potential. RGZ treatment also induced upregulation of Beclin-1 and LAMP-1, two proteins involved in different steps of the autophagic process (42). Another more recent report related induction of autophagy with cell death in ACC (43). In fact, it was shown that etoposide treatment inhibited the growth of the Y1 cells (murine adrenocortical tumor cell line) by inducing cellular senescence rather than apoptosis. Further experiments revealed that etoposide treatment triggered the signaling cascade involving DNA-PK-Chk2 for activating autophagy, inducing primary ciliogenesis and multiple centrosomes which is followed by cell senescence. Interestingly, the authors hypothesize that, upon etoposide treatment, Y1 cells start to grow cilia for cell cycle arrest and possibly, to gain the ability to become drug-resistant cells. This hypothesis should be further investigated in the future in order to improve therapeutic efficacy (43).

Autophagy induction was also reported in PCC treatment. Using biochemical and biophysical methods, Dong et al. argued that treatment with maslinic acid (MA), a natural compound with anti-tumoral effect in different models, induced autophagy in PC-12 cells (44). The authors verified that MA treatment promoted LC3-I/LC3-II conversion and disrupted the interaction between BCl-2 and Beclin-1, allowing the recruitment of autophagy positive regulators. It would be interesting to perform further studies in order to evaluate the dynamics of the autophagic flux following the treatment with maslinic acid, namely p62 accumulation or co-treatment with autophagy modulators such as wortmannin or bafilomycin A1, as referred in section 2.

The promotion of autophagy in PCC treatment was also associated to Notch1 signaling. Notch1 signaling pathway has been referred as an important target for chemotherapy in PCC, however, the mechanistic effect of this modulation remains unknown (45, 46). Bo Li et al. using a tetracycline-inducible system which increase the expression of Notch1 intracellular domain (NICD), showed that high levels of NICD induced apoptosis, inhibited PC12 cells proliferation and increased the expression of LC3, Beclin-1, ATG5 and ATG7 (47). The authors argued that these results indicated that NCID overexpression may be a promising potential therapy for PCC. However, the cross-talk between apoptosis and autophagy was not further analyzed. Therefore, further studies about this association are required in order to better characterize this treatment option.

We described above that the comprehension of the role of autophagy in adrenal tumors context is challenging due to the dual role of autophagy induction. Indeed, in both ACC and PCC, the referred studies showed that autophagy activation promoted: i) a protective (pro-survival) mechanism of tumor cells regarding chemotherapeutic drugs resulting in treatment failure and ii) a tumor cell death mechanism induced by therapy resulting in treatment success. Understanding this may be even more challenging since autophagy reduction may also be associated with cell death process. A study aiming to investigate the cytotoxic effect of ursolic acid (UA) in PC-12 cells, revealed that treatment with this natural compound induced the accumulation of both LC3-II and p62 proteins, indicating a blockage in the autophagic flux, and consequently inhibition of autophagy (48). In addition, the expression of BCl-2 was decreased and caspase-3 was found to be activated, suggesting the induction of apoptosis. Taken together, these results suggested that in PC-12 cell line, the induction of cell death by UA treatment is triggered by a combination of two mechanisms: induction of apoptosis and impairment of autophagy. Similarly, it was shown that apoptosis triggered by graphene oxide in PC-12 cells was dependent of autophagic flux blockage (namely p62/SQSTM1 complex) and lysosomal dysfunction (49).

## 5. The Role of Autophagy in Steroidogenesis in Adrenal Gland

Steroidogenesis is a relevant cellular process which occurs in the cortex of adrenal gland. A cell is classified as “steroidogenic” if the enzyme P450scc is present, catalyzing the first reaction of steroidogenesis (i.e., the conversion of cholesterol to pregnenolone) (50, 51). As referred above (section 1), the adrenal cortex is responsible for the production of three main subclasses of steroid hormones: i) glucocorticoids (mainly cortisol), ii) mineralocorticoids (mainly aldosterone), and iii) adrenal androgens and their precursors (mainly DHEA, DHEAS, and androstenedione). Following the transport of intracellular cholesterol into the adrenal cortex under stimulation of ACTH, steroidogenesis involves the collaborative action of a series of enzymes (52). Interestingly, the inhibition of autophagy was shown to decrease lipid droplets, triglycerides, and cholesterol in Leydig and adrenocortical cells suggesting that autophagy or lipophagy (autophagic degradation of lipid droplets) may regulate lipid homeostasis in both steroidogenic cell types (53). Indeed, the role of autophagy in steroidogenic cells was reported years before, in 1968, following observation of autophagic vacuoles in the interstitial cells of Guinea pig testis (54). Later studies showed the formation of autophagosomes containing mitochondria in Leydig cells and associated autophagy with testosterone production (55, 56).

The regulation of the dynamics of lipid droplets in steroidogenic cells by autophagy may have particular relevance in the improvement of ACC therapeutic options. Most of ACCs produce excessive amounts of steroid hormones (or their precursors) and therefore, inhibition of steroidogenesis needs to be considered in ACC treatment. Treatment with sunitinib, a multi-tyrosine kinase inhibitor, was found to decrease the proliferation of adrenocortical carcinoma cells and to block the steroidogenesis (57). Nevertheless, in this study the modulation of autophagy was not assessed. Interestingly, in other tumor models, such as clear cell ovarian carcinoma and pancreatic neuroendocrine tumors, the inhibition of autophagy improved the efficacy of sunitinib treatment (58, 59). Moreover, the inhibition of autophagy enhanced the sunitinib-induced cytotoxicity in PC-12 cells, as described in section 4. Based on these results, we argue that the effect of the co-treatment of sunitinib with an autophagy inhibitor should be evaluated in ACC. We hypothesize that in this context, the inhibition of autophagy may promote a dual effect: improvement of sunitinib-induced proliferation reduction and blockage of steroidogenesis.

The interplay between steroidogenesis and autophagy in ACC was also demonstrated by the modulation of the transcription factor estrogen-related receptor alpha (ERRα). ERRα is expressed in normal adult adrenal gland and regulates the expression of enzymes involved in steroidogenesis (60). Importantly, ERRα seems to be upregulated in ACC compared to normal adrenal tissue and adrenal adenomas and it is a downstream target of different pathways that are altered in ACC such as IGFII/IGF1R, β-catenin, Wnt and ESR1 signaling pathways (61). XCT790, an inverse agonist of ERRα, was shown to reduce H295R cell growth both *in vitro* and *in vivo* (62). XCT790 treatment altered cell cycle profile but did not trigger apoptosis. Instead, the alterations in cell cycle profile induced by XCT90 treatment were followed by incomplete autophagy and cell death by necrosis. Moreover, in ACC cells, ERRα depletion after XCT790 treatment caused a reduction of mitochondrial function promoting the activation of a number of cellular mechanisms that also result in tumor cell death. Altogether, these findings suggest that ERRα is a promising target for the therapy of ACC and impairment of autophagy plays a role in this mechanism (62).

## 6. Crosstalk Between Autophagy and Extracellular Vesicles Biogenesis in Adrenal Tumors

Extracellular vesicles (EVs) have been largely studied due to their potential in diagnosis, prognosis, monitoring and treatment of different cancers (63, 64). EV is a generic term for all types of lipid bilayer-delimited particles that cannot replicate. EVs are released virtually by all type of cells and they are present in different biological fluids such as blood and urine (65). According to their biogenesis, two main classes of EVs have been described: exosomes and microvesicles. Typically, exosomes are produced along the endocytic pathway (through multivesicular body [MVB] maturation) and range in size from 30 to 150 nm. Instead, microvesicles result from the budding and blebbing of plasma membrane, ranging in size between 50 to 1000 nm (66). Interesting findings suggest that, among other factors, the regulation of the production and secretion of EVs *via* endocytic pathway is associated with cellular autophagic status.

In the recent years, some works have been studying the EVs released by adrenal tumor cells. The EVs of patients with PCC and paraganglioma were shown to contain double stranded DNA (dsDNA) that can reflect the mutation status of susceptible genes. The authors argued that EVs- associated dsDNA can be one of the most effective methods for somatic mutation screening (67). Furthermore, the EV-associated miRNAs were found differentially expressed in different non-functioning and cortisol-producing ACT (68). For example, EV-associated hsa-miR-483-5p appears to be a promising minimally invasive biomarker in the preoperative diagnosis of ACC (69). To the best of our knowledge, none of the studies of EVs in adrenal tumor context addressed the fundamental issues of EVs release and uptake by donor and recipient cells, respectively. We argue that this knowledge will be of outmost importance since it will allow the use of EVs as non-invasive diagnostic, prognostic and therapeutic tools in adrenal tumors.

The decision of cellular fate either for autophagy or for EVs production has been studied and the endocytic pathway seems to play a major role in this cross-talk, namely towards early endosome (**Figure 1**). Firstly described in the 1980s, the intracellular compartments called endosomes (70) were shown to differ on kinetic, molecular, and morphological criteria (71). In endocytic pathways, early endosomes are the first to be generated and receive almost instantly the cargo from endocytosis or from membrane fusion. Upon the incorporation into an early endosome, the cargo is sorted into different pathways, therefore early endosome is also termed as the “sorting” endosome (72). Early Endosome Antigen 1 (EEA1) is a typical marker of early endosome. Sbiera et al. aiming to study the mechanism of action of mitotane revealed that besides its connection to mitochondrial membranes, mitotane also accumulated in membranes of early endosomes (identified by the expression of EEA-1) (73). This finding is not unexpected due to lipophilic nature of mitotane. Nevertheless, we have to recognize that the effect of mitotane treatment in the fate of endocytic pathway and its impact for autophagy status in adrenal tumor cells requires further investigation.

Early endosomes undergo homotypic fusion being matured into late endosomes/MVB (71, 74). Following maturation, the late endosome/MVB may fuse with the plasma membrane to release EVs or be fated for cargo degradation. The regulation of the balance between MVB fate to secretion and degradation remains unexplored (75). It seems that the destiny of the MVB may change due to alterations in cellular conditions. For example, starvation induces MVB degradation by fusion with autophagosomes (autophagy), resulting in a decrease in the number of EVs released (76). The fusion of endosomes/MVBs with the autophagosome requires further study.

The initial studies showed no labelling of endosomal proteins in early autophagosomes (77), nevertheless, recent findings showed that the endocytic pathway may contribute to phagophore formation and expansion (78). Different families of proteins are involved in the regulation of EVs production by endocytic pathway, such as endosomal sorting complexes required for transport (ESCRT) or Rab family. Interestingly, a recent proteomic study performed by liquid chromatography–tandem mass spectrometry (LC–MS/MS) of 45 formalin-fixed paraffin-embedded ACC tissues showed that in the top 20 of differentially expressed proteins (DEPs) comparing stage 1–2 vs. 3–4 tumors, two DEPs belong to Rab family: RABL3 e RABAC1 (79). RABAC1 was previously found in late endosomes (80). The association of autophagy and endocytic pathway was also referred in other study. RGZ treatment of ACC cells induced autophagy, increased Beclin-1 levels and also LAMP-1 (marker of late endosome/lysosome) (42). Further studies about the impact of those proteins in EVs release and autophagy status in ACC will enhance both basic understanding and translational potential of EVs in this malignancy.

## 7. Final Remarks

Early diagnosis and treatment of malignant adrenal tumors still require deeper advances. Regardless of novel discoveries, curative options are still highly limited and the poor outcome has not improved significantly over the past 40 years. It has become increasingly clear that deciphering the molecular mechanisms underlying the pathophysiology and treatment possibilities of both ACC and PCC is a burning need for precision medicine.

Autophagy comprehension and modulation has been referred as an important strategy for the management of endocrine tumors. Regarding malignant adrenal tumors in particular, unraveling the molecular mechanisms regulating autophagic flux is an emerging field. In the present review, we illustrated two main features of autophagic process in adrenal tumors already described in other tumor models: i) the dual role associated with pro-survival or pro-death outcome, depending on context and ii) the interplay of autophagy with other cellular pathways, namely endocytic and apoptotic pathway.

**Figure 2:**
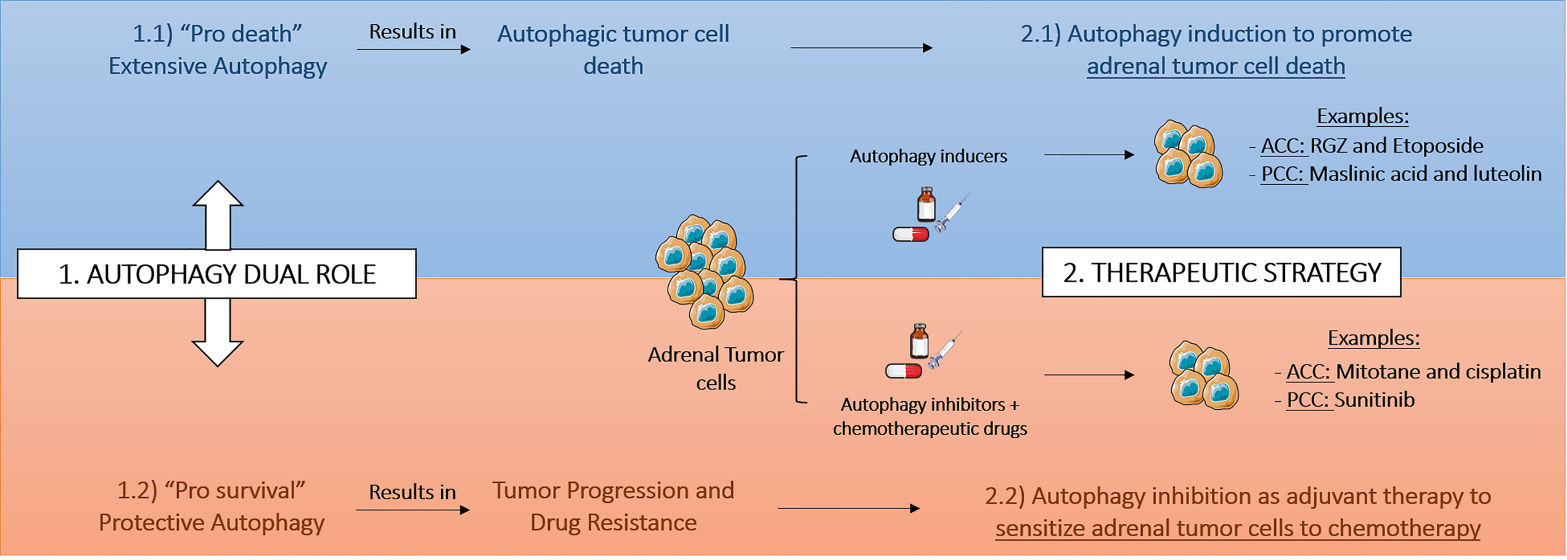
Therapeutic strategies based on autophagy modulation in adrenal tumors. The therapeutic interest and consequently the therapeutic strategy based on autophagy modulation in adrenocortical cancer (ACC) and pheochromocytoma (PCC) depends on pro-survival or pro-death tumoral context. RGZ, rosiglitazone.

The studies performed on adrenal tumors have been showing that the therapeutic interest of autophagy modulation in ACC and PCC is influenced by pro-survival or pro-death context of autophagy (**Figure 2**). Similar to other models, such as breast cancer, the modulation of autophagy may be dependent on the stage of disease or on the treatment course (81). Drugs, such as etoposide or RGZ in ACC or maslinic acid and luteolin in PCC, induce extensive autophagy, mediating the so-called autophagic cell death in the cancer cells (**Table 1**). In other context, autophagy promoted by chemotherapy treatment or due to tumor microenvironment may result in a cytoprotective effect and therefore trigger drug resistance and tumor relapse. Interestingly, mitotane and cisplatin in ACC model or sunitinib in PCC model were shown to promote better effects on reduction of cellular proliferation or induction of apoptosis with concomitantly autophagy inhibition. Indeed, autophagy might act as a survival mechanism for tumor hypoxic cells through recycling of cellular constituents. The hypoxia risk signature was recently suggested to be able to predict prognosis and evaluate the tumor immune microenvironment of ACC (82) and also the long common history between hypoxia and PCC (83), indicate to us that in adrenal tumorsit would be interesting to study the hypoxia status and relate it with improved therapeutic strategies using autophagy modulation.

**Table 1:** Autophagy modulation in adrenal tumors.

Adrenal Tumor Type	Featured findings	Reference
ACC	Positive expression of Beclin-1 in ACC tissues was significantly lower when compared with normal tissues. Negative expression of Beclin-1 was significantly correlated with advanced stage, regional lymph, node metastasis, increasing T stage, and poor differentiation.	(31)
ACC	Proportion of positive LC3B in isolated single positive cells was higher in ACC than in ACA.	(30)
ACC	Combination therapy of mitotane with chloroquine (an autophagy inhibitor) promoted higher levels of cleaved PARP, suggesting higher apoptosis induction, when compared with mitotane treatment alone.	(36)
ACC	Tauroursodeoxycholic acid treatment alleviated endoplasmic reticulum (ER) stress, induced autophagy and inhibited apoptosis of ACC cells.	(37)
ACC	Concomitant treatment of cisplatin with chloroquine (autophagy inhibitor) increased the apoptosis rate of ACC cells when compared with cisplatin monotherapy. These results were confirmed *in vivo*.	(31)
ACC	Rosiglitazone (RGZ) inhibited the growth of ACC cells, activating the AMPK pathway, resulting in cellular vacuolization and enhanced autophagy. RGZ treatment also induced upregulation of Beclin-1 and LAMP-1.	(42)
ACC	Etoposide treatment inhibited the growth of ACC cells by inducing cellular senescence and by triggering the signaling cascade involving DNA-PK-Chk2 for activating autophagy.	(43)
PCC	P62 positivity was shown to be predictive of shorter overall survival in PCC patients.	(30)
PCC	Downregulation of ATG13 promoted an increase in apoptosis and a reduction in proliferation of PCC cells following treatment with sunitinib.	(40)
PCC	Treatment with luteolin induced autophagy and apoptosis through activation of ER stress sensors in PC12 cells.	(38)
PCC	Maslinic acid, a natural compound with anti-tumoral effect in PCC cells, promoted LC3-I/LC3-II conversion and disrupted the interaction between BCl-2 and Beclin-1, inducing autophagy in those cells.	(44)
PCC	Increased expression of Notch1 intracellular domain induced apoptosis, inhibited proliferation and increased the expression of LC3, Beclin-1, ATG5 and ATG7 in PCC cells.	(47)
PCC	Ursolic acid (UA) promoted PCC cell death by inducing the accumulation of both LC3-II and p62 proteins, indicating a blockage in the autophagic flux, and consequently inhibition of autophagy. UA treatment decreased BCl-2 levels and activated caspase-3, suggesting the induction of apoptosis.	(48)
PCC	Apoptosis induced by graphene oxide in PCC cells was dependent of autophagic flux blockage (namely p62/SQSTM complex) and lysosomal dysfunction.	(49)

ACC, adrenocortical carcinoma.

PCC, pheochromocytoma.

The findings reviewed here warrant further investigation in order to better clarify the clinical relevance of autophagy modulation in adrenal tumors. It is noteworthy that studies performed in tissue samples from patients were still a minority and therefore, in the future, these will be required. In addition, it is important to refer that the comprehension of adrenal tumorigenesis, tumor progression or drug response is also related with understanding of the mechanisms involved in hormone production and secretion. Due to this complexity, a reliable *in vitro* model which challenges all these molecular approaches is difficult to establish. Furthermore, *in vitro* studies focused in unraveling the role of autophagy in adrenal tumors will require further details. Studying the autophagic flux, namely by silencing core proteins, such as ATG family, co-treatment with known autophagic modulators and evaluation of both LC3-II and p62 levels will strength the conclusions obtained. Due to these studies requiring further detailed mechanistic knowledge, among more than one hundred of the clinical trials conducted or ongoing to explore the efficacy of autophagy modulators to reduce the tumor growth and potentiate the anti-cancer effects of conventional therapy, there is none including adrenal tumors (84). Currently, there are 16 ACC studies and 37 PCC studies actively enrolling patients, as seen on clinicaltrials.gov. Few of these trials are related to immunotherapy with anti-PD-1 antibodies. It is known that autophagy regulates both positively and negatively PD-L1 expression on cancer cells (85)and therefore this should also be further explored in adrenal tumors context. This is even more relevant since different drugs which are being used in those clinical trials were demonstrated to modulate autophagy in adrenal tumors *in vitro* and *in vivo* such as etoposide or mitotane.

Only a detailed evaluation will allow to find novel targets and strategies to improve both diagnosis and treatment of ACC and PCC, and we postulate that it will be essential to elicit or modulate the regulation of autophagy in different stages of ACC and PCC tumorigenesis, tumor growth and tumor invasion.

## Author Contributions

DP, SP and DS contributed to the conception, literature review and writing of the manuscript. All authors contributed to the article and approved the submitted version.

## Funding

This study was funded by the Foundation for Science and Technology (FCT) through the following funds: PTDC/MEC-ONC/31384/2017, UIDB/00215/2020, UIDP/00215/2020 and LA/P/0064/2020.

## Conflict of Interest

The authors declare that the research was conducted in the absence of any commercial or financial relationships that could be construed as a potential conflict of interest.

## Publisher’s Note

All claims expressed in this article are solely those of the authors and do not necessarily represent those of their affiliated organizations, or those of the publisher, the editors and the reviewers. Any product that may be evaluated in this article, or claim that may be made by its manufacturer, is not guaranteed or endorsed by the publisher.
